# Genetic diversity and gene differentiation among ten species of Zingiberaceae from Eastern India

**DOI:** 10.1007/s13205-013-0166-9

**Published:** 2013-09-01

**Authors:** Sujata Mohanty, Manoj Kumar Panda, Laxmikanta Acharya, Sanghamitra Nayak

**Affiliations:** Centre of Biotechnology, Siksha ‘O’ Anusandhan University, Bhubaneswar, 751003 Odisha India

**Keywords:** Zingiberaceae, Genetic diversity, RAPD, ISSR, SSR

## Abstract

In the present study, genetic fingerprints of ten species of Zingiberaceae from eastern India were developed using PCR-based markers. 19 RAPD (Rapid Amplified polymorphic DNA), 8 ISSR (Inter Simple Sequence Repeats) and 8 SSR (Simple Sequence Repeats) primers were used to elucidate genetic diversity important for utilization, management and conservation. These primers produced 789 loci, out of which 773 loci were polymorphic (including 220 unique loci) and 16 monomorphic loci. Highest number of bands amplified (263) in *Curcuma caesia* whereas lowest (209) in *Zingiber cassumunar*. Though all the markers discriminated the species effectively, analysis of combined data of all markers resulted in better distinction of individual species. Highest number of loci was amplified with SSR primers with resolving power in a range of 17.4–39. Dendrogram based on three molecular data using unweighted pair group method with arithmetic mean classified all the species into two clusters. Mantle matrix correspondence test revealed high matrix correlation in all the cases. Correlation values for RAPD, ISSR and SSR were 0.797, 0.84 and 0.8, respectively, with combined data. In both the genera wild and cultivated species were completely separated from each other at genomic level. It also revealed distinct genetic identity between species of *Curcuma* and *Zingiber*. High genetic diversity documented in the present study provides a baseline data for optimization of conservation and breeding programme of the studied zingiberacious species.

## Introduction

*Zingiber* and *curcuma* are two interesting genus of Zingiberaceae mostly contain spice plants with very high medicinal value. Among them few species are being cultivated but majority of them are wild in nature. Thus, these species are being depleted from nature due to extensive collection and habitat destruction. Strategic management and planning for their conservation is still far away from reality. Some species are still unknown due to their unavailability and extensive use by the tribal people for medicine as well as spice. The knowledge of genetic variability is a pre requisite to study the evolutionary history of a species, as well as for other studies like intraspecific variation, genetic resource conservation etc. (Islam et al. 2007). Hence, genetic diversity and gene differentiation through molecular marker analysis are essential for their taxonomic relationship evaluation, conservation and sustainable utilization.

For proper conservation programme it is essential to characterize the plants genetically. Number of molecular markers is being regularly used for studying genetic relations, population genetics, genetic characterizations in different plant groups and cultivars. The molecular markers are not influenced by the external environmental factor unlike that of morphological markers and hence accurately testify the genetic relationship between and among plant groups. Molecular markers like RAPD, ISSR and SSR are being used regularly for genetic diversity assessment as a thorough knowledge of the level and distribution of genetic variation is essential for conservation (Dreisigacker et al. 2005; Sharma et al. 2008; Naik et al. 2010; Das et al. 2011). PCR-based DNA fingerprinting techniques like RAPD, ISSR and SSR are proven to be very informative and cost-effective techniques in many plant species as these primers do not require prior knowledge of a species genetics (Williams et al. 1990; Zeitkiewicz et al. 1994; Lee et al. 2007).

Many workers have been reported the genetic diversity among zingiber and curcuma species (Das et al. 2011; Jatoi et al. 2006; Syamkumar and Sasikumar 2007) but the studied species are area specific based on their availability in that region. Also, less is known about the genetic relationship among cultivated and wild species which is the main reason for extinction of wild but important species having future drug yielding potential. Studies have been attempted based on morphological, biochemical and anatomical characterization in *Zingiber* and *Curcuma* species (Jiang et al. 2006; Zhou et al. 2007; Paramasivam et al. 2009), but relying on morphological and biochemical characters it has its own limitations as they are always not completely represent the genetic structure (Noli et al. 1997). Molecular profiling of zingiberaceous species is still in an emerging stage. Reports were restricted to specific species and species within a single genus. Thus the present work is an attempt to study the genetic relationship existing within and among two different genera i.e., *Zingiber* and *Curcuma*.

The objective of the present study is to examine the level of genetic diversity among and within two populations of Zingiberaceae using DNA-based molecular markers like RAPD, ISSR and SSR to give baseline knowledge for optimization of combining strategies for efficient management of both the population.

## Materials and methods

### Plant material and DNA extraction

The present investigation deals with ten species of Zingiberaceae from eastern India and was collected from both Odisha and West Bengal. Among them *Z. officile* and *C. longa* are cultivated varieties and rest species were collected from wild habitat. They were maintained in the greenhouse of Centre of Biotechnology of Siksha ‘O’ Anusandhan University. Genomic DNA was isolated from frozen leaf samples by grinding with a mortar, pestle in extraction buffer (0.1 M Tris–HCl pH 7.5, 0.25 M NaCl, 25 mM EDTA pH 8.0, 10 % CTAB), and incubated at 65 °C for 10 min. It was thrice treated with phenol: chloroform: isoamyl alcohol (25:24:1) for removal of non-nucleic acid compounds. DNA was precipitated using isopropanol and resuspended in 100 μl of 10 mM Tris, pH 8.0 with 10 μg RNaseA. The quality and quantity of the DNA was determined with a Thermo Scientific UV–Vis spectrophotometer. The sample DNA was diluted as 25 ng μl^−1^ for RAPD and ISSR analysis.

### Molecular marker analysis

Nineteen RAPD primers (Operon Technologies, Alemada, USA), eight ISSR and eight SSR primers (Bangalore Genei, Bangalore, India) were used for PCR amplification. Based on previous results, good resolution and reproducibility ability, all RAPD, ISSR and ISSR primers were selected out of several primers utilized during screening. In RAPD, PCR was performed at an initial temperature of 94 °C for 5 min for complete denaturation. The second step consisted of 42 cycles having three ranges of temperature, i.e., at 92 °C for 1 min for denaturation of template DNA, at 37 °C for 1 min for primer annealing, and at 72 °C for 2 min for primer extension, followed by running the samples at 72 °C for 7 min for complete polymerization. For ISSR, the same temperature profile was followed, but the primer annealing temperature was set at 5 °C lower than the melting temperature. The PCR products obtained from RAPD were analyzed in 1.5 % agarose gel whereas the ISSR products were analyzed in 2 % agarose gel stained with ethidium 15-μl of PCR products from RAPD and ISSR markers were combined with 2-μl of a loading buffer (0.4 % Bromo-phenol Blue, 0.4 % xylene cyanole and 5 ml of glycerol) and were analyzed directly on 1.5 % agarose gels in 0.5X TBE buffer. Electrophoresis was done for about 3 h at 60 volts. 100 bp ladder (MBI Fermentas) was used to compare the molecular weights of amplified products. Visualization of the amplified bands was done by UV transilluminator (Bgenei, Bangalore, India).

The detection of microsatellite polymorphism was performed using seven SSR markers from the eight SSR markers characterized by Lee et al. (2007). The SSR amplification condition was as follows: an initial hot start and denaturing step at 93 °C for 3 min followed by 35 cycles of a 1 min denaturation at 93 °C, a 1 min annealing at 55 °C, and a 1 min primer elongation at 72 °C. A final extension step at 72 °C for 5 min was performed. The PCR amplified products were resolved in a 12 % non-denaturing polyacrylamide gel. Electrophoresis was done for about two and half hours at 120 volts. 50 bp ladder (MBI Fermentas) was used to compare the molecular weights of amplified products.

### Data analysis

Bands generated by all markers were compiled into a binary data matrix based on the presence (1) or absence (0) of the selected band. The Primer Index was calculated from the polymorphic index. Polymorphic index of polymorphic information content (PIC) was calculated as per the formula of Roldan-Ruiz et al. (2000). Jaccard’s coefficient of genetic similarity was calculated using the binary data matrix between all accessions. Similarity coefficient values estimated were used to construct a dendrogram (cluster diagram) using the method of unweighted pair group with arithmetic averages (UPGMA) and principal co-ordinate analysis (PCA) was also carried out using NTSYSpc ver.2.2 software (Rohlf 1997).

## Results

### RAPD polymorphism

**Fig. 1:**
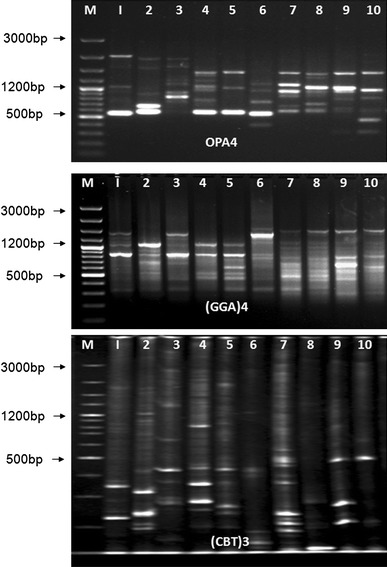
RAPD, ISSR and SSR banding pattern in 10 species of Zingiberaceae with primer OPA4,(GGA)4 and (CBT)3 (M = gene ruler 100 bp ladder. Lane 1–10 represents, *Z. officinale*, *Z. rubens, Z.zerumbet, Z. chrysanthum, Z. clarkii, Z. cassumunar, C. longa, C. amada, C. aromatica, C. caesia*

Twenty-five random decamer oligonucleotide primers were used for the present work and out of them 19 primers were selected and proved to be informative. A total of 317 bands amplified, were all polymorphic in nature. Among all the primers highest number of bands (29) were amplified in primer OPA4 which were in the range of 320–2,100 bp. Lowest number of bands (3) was amplified in primer OPD12 in the range of 850–1,031 bp. Average number of bands per primer was found to be 16.7. The largest amplicons (>3,000 bp) were amplified with OPA7, OPC2, OPD18 and OPAF5 primer and the smallest (200 bp) with OPA18, OPN16, and OPAF5 primer (Table 1) (Fig. 1). Among all the polymorphic bands amplified 111 numbers of bands were found to be unique. Highest number of unique bands (12) was amplified in primer OPA7 in and lowest (2) in primer OPD7, OPD12, respectively. The resolving power of the primers was varied from 11.27 to 0.72 where the primer OPD20 had maximum resolution power (11.27) and the primer OPD12 had minimum resolution power (0.727). However, the RAPD primer index was maximum (8.264) in case of primer OPA4 and minimum (0.628) in primer OPD12. The RPI value revealed that the primer OPA4 was the best for species segregation at nuclear DNA level. Jaccard’s coefficient showed that the species *C. amada* and *C. aromatica* were most closely related with a similarity value of 0.29 and *Z. cassumunar* and *C. amada* were most remotely placed with a similarity coefficient of 0.132. Between any two species the average similarity coefficient was calculated to be 0.121.

**Table 1:** Details of RAPD and ISSR analysis in 10 species of Zingiberaceae

Markers	Primer	Primer sequence	Approx fragment Size (bp)	Total bands	Unique bands	Resolving power	Primer index
RAPD	OPA4	5′AATCGGGCTG3′	320 to 2,100	29	10	10.72	8.26
	OPA7	5′GAAACGGGTG3′	300 to >3,000	21	12	7.09	5.45
	OPA9	5′GGGTAACGCC3′	600 to 2,100	12	6	4.00	3.07
	OPA18	5′AGGTGACCGT3′	200 to 2,250	25	8	9.81	7.33
	OPC2	5′GTGAGGCGTC3′	500 to >3,000	22	8	10.36	7.07
	OPC5	5′GATGACCGCC3′	900 to 2,500	14	4	5.81	4.36
	OPC11	5′AAAGCTGCGG3′	450 to 2,100	17	3	9.27	6.11
	OPD3	5′GTCGCCGTCA3′	400 to 1,950	13	6	4.90	3.66
	OPD7	5′TTGGCACGGG3′	400 to 2,200	17	2	9.09	6.34
	OPD8	5′GTGTGCCCCA3′	500 to 2,800	18	10	6.90	4.69
	OPD12	5′CACCGTATCC3′	850 to 1,031	3	2	0.72	0.62
	OPD18	5′GAGAGCCAAC3′	450 to >3,000	16	4	6.90	4.86
	OPD20	5′ACCCGGTCAC3′	400 to 3,000	24	6	11.27	7.90
	OPN4	5′GACCGACCCA3′	500 to 2,200	16	3	8.54	5.58
	OPN16	5′AAGCGACCTG3′	200 to 2,100	18	11	6.54	4.72
	OPN18	5′GGTGAGGTCA3′	400 to 1,800	14	3	5.81	4.26
	OPAF5	5′CCCGATCAGA3′	200 > 3,000	15	5	6.90	4.36
	OPAF14	5′GGTGCGCACT3′	400 to 3,000	13	4	4.90	3.80
	OPAF15	5′CACGAACCTC3′	550 to 2,000	10	4	3.45	2.71
	Total		–	317*	111	–	–
	Mean		–	16.7	5.8	6.99	5
	Range		200 to >3,000	3–29	2–12	0.72–11.27	0.62–8.26
ISSR	SPS 1	(GAC)5	200 to 1,200	16**	2	14.72	4.92
	SPS 2	(GTGC)4	250 to 2,000	17	7	10.90	5.52
	SPS 3	(GACA)4	300 to 3,000	22	4	16.54	8.16
	SPS 4	(AGG)6	325 to 1,700	21	6	13.27	7.20
	SPS 5	(GA)9T	350 to 2,000	19	2	12.00	6.64
	SPS 6	T(GA)9	325 to 1,400	17	6	10.54	6.67
	SPS 7	(GTG)5	250 to 1,200	15**	6	11.09	4.79
	SPS 8	(GGA)4	300 to 1,800	20**	3	18.36	7.93
	Total		–	147	36	–	–
	Mean		–	18.37	4.5	13.42	6.47
	Range		200 to >3,000	15–22	2–7	10.54–18.36	4.79–8.16

* All are polymorphic bands, ** contain one monomorphic band, rest are all polymorphic

### ISSR polymorphism

Out of 10 ISSR primers, 8 primers resulted in the amplification of 147 fragments of which 3 were monomorphic bands and 144 were polymorphic bands which again includes 36 unique bands. The bands were amplified in the range of 200–3,000 bp (Fig. 1). The primer (GACA)_4_ produced maximum number of bands (22), while the primer (GTG)_5_ resulted in amplification of 15 loci only (Table 1). Maximum number of polymorphic bands (22) was found in primer (GACA)_4_ and minimum (15) bands amplified in primer (GTG)_5_. Monomorphic bands were found in primer (GTG)_5_, (GAC)_5_ and (GGA)_4_ whereas unique bands were found in all primers. Among these ISSR primers, maximum resolving power was obtained for (GGA)_4_ (18.364) and the minimum was for T(GA)_9_ (10.545) with an average of 13.42. Maximum primer index was calculated for (GACA)_4_ and minimum for (GTG)_4_ with an average of 6.47. Jaccard’s coefficient showed that the species *C. caesia* and *C. aromatica* were most closely related with a similarity value 0.67. Between any two species the average similarity coefficient was calculated at 0.311.

### SSR polymorphism

Eight SSR primers were used for the present work. The primer combinations had amplified 325 loci among which 13 were found to be monomorphic in nature and the rest were polymorphic (Table 2) (Fig. 1). From the 312 polymorphic bands amplified, 73 were found to be unique. Maximum number of 52 bands was resolved for the primer CBT04 and the minimum 21 for CBT09. The average number of bands amplified for primer was 40.62. Bands resolved between 3,000 and 20 bp were taken into consideration for the present investigation. Maximum number of polymorphism was found in primer CBT04 (98 %) and minimum in primer CBT07 (91 %). The resolving power was maximum for the primer CBT04 (39.455) and minimum for primer CBT09 (17.455) with an average of 27.97. However, the primer index was found to be highest in the primer CBT04 (20.231) and lowest in primer CBT09 (7.074) with an average of 13.61. Jaccard’s coefficient showed that the species *Z. rubens* and *Z. zerumbet* were most closely related with a similarity value 0.476 and *C. caesia* and *Z. cassumunar* were most remotely placed with a similarity coefficient of 0.17. Average similarity coefficient was calculated at 0.296.

**Table 2:** Details of SSR analysis in 10 species of Zingiberaceae

Primer	Primer sequence	Approx fragment Size(bp)	Total bands	Polymorphic Bands	Pol (%)	Monomorphic bands	Unique bands	Resolving power	SSR primer index
CBT-02	F:TCCTCCCTCCCTTCGCCCACTG R:CGATGTTCGCCATGGCTGCTCC	180 to 2,500	47	44	93.6	3	7	38.18	16.82
CBT-03	F:ATCAGCAGCCATGGCAGCGAC R:AGGGGATCATGTGCCGAAGGC	100 to 3,000	47	46	97.8	1	4	35.63	18.44
CBT-04	F:ACCCTCTCCGCCTCGCCTCCTC R:CTCCTCCTCCTGCGACCGCTCC	<100 to 2,900	52	51	98.1	1	5	39.45	20.23
CBT-05	F:CTCTGTCTCCTCCCCCGCGTCG R:TCAGCTTCTGGCCGGCCTCCTC	<100 to 3,000	49	48	97.9	1	13	36.72	16.13
CBT-06	F:GCCTCGAGCATCATCATCAG R:ATCAACCTGCACTTGCCTGG	<100 to 3,000	42	40	95.2	2	20	18.36	10.51
CBT-07	F:CGATCCATTCCTGCTGCTCGCG R:CGCCCCCATGCATGAGAAGAG	<100 to >3,000	34	31	91.2	3	15	18.36	8.33
CBT-08	F:CAGCAGATTTTTGCTCCG R:GTCGCGTTCGTGGAAAT	<100 to >3,000	33	32	96.9	1	6	19.63	11.37
CBT-09	F:AGGGGGCAGTGGAGAG R:ACGTTCCTGCACTTGACG	<100 to 1,600	21	20	95.2	1	3	17.45	7.07
Total		–	325	312	–	13	73	–	–
Mean		–	40.62	39	95.7	1.6	9.1	27.97	13.6
Range		<100 to >3,000	21–52	20–51	91–98	1–3	3–20	17.4–39.4	7.07–20.23

### Combined marker analysis

**Fig. 2:**
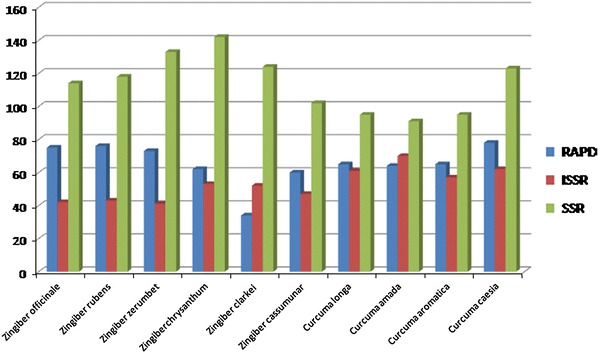
Total number of bands amplified in ten species of zingiberaceae with RAPD, ISSR and SSR primers

Total number of bands amplified with all markers in ten species is 2317 (Fig. 2) with an average of 231.7 per species. Highest number of bands amplified (263) in *Curcuma caesia* whereas lowest (209) in *Zingiber cassumunar*. Average number of bands amplified in all species is 16, 18 and 30 with RAPD, ISSR and SSR, respectively. Jaccard similarity showed that the species *C. aromatica* and *C. amada* were closely related with a similarity value of 0.4. Most distantly placed species were *C. aromatica* and *Z. cassumunar* with a similarity value of 0.132. The average similarity was found to be 0.19 (Table 3).

**Table 3:** Jaccard’s similarity coefficient among 10 species of Zingiberaceae for RAPD, ISSR and SSR

	*Z. officinale*	*Z. rubens*	*Z. zerumbet*	*Z. chrysanthum*	*Z. clarkei*	*Z. cassumunar*	*C. longa*	*C. amada*	*C. aromatica*	*C. caesia*
*Z. officinale*	1.0000									
*Z. rubens*	0.1754	1.0000								
*Z. zerumbet*	0.2183	0.1863	1.0000							
*Z. chrysanthum*	0.1787	0.2487	0.2081	1.0000						
*Z. clarkei*	0.2012	0.2197	0.2012	0.3019	1.0000					
*Z. cassumunar*	0.1949	0.1463	0.2139	0.2094	0.2025	1.0000				
*C. longa*	0.1539	0.1577	0.1914	0.1991	0.1848	0.1691	1.0000			
*C. amada*	0.1429	0.2036	0.1781	0.1644	0.2270	0.1461	0.2757	1.0000		
*C. aromatica*	0.1652	0.1741	0.1333	0.1614	0.2174	0.1324	0.2796	0.4091	1.0000	
*C. caesia*	0.1458	0.1542	0.1447	0.1717	0.1553	0.1342	0.2793	0.3139	0.3810	1.0000

The dendrogram constructed through SHAN clustering and UPGMA analysis using Jaccard’s similarity coefficient by RAPD, ISSR and SSR divided it into two clusters consisting *Zingiber* and *Curcuma* species. The dendrogram obtained presents two main clusters with ten species. The first cluster was again divided into two sub-clusters, the first having species *Z. chrysanthum, Z. clarkei* and *Z. rubens* whereas the second subcluster was consisting of *Z. officinale* cv. Suprava, *Z. zerumbet* and *Z. cassumunar,* respectively. The second main cluster consists of *C. aromatica, C. longa*, *C. amada* and *C. caesia.* The two clusters had bootstrap value of 100 which proved its stability. The two subclusters were also stable having bootstrap value 72 and 88, respectively. Mantle matrix correspondence test revealed high matrix correlation in all the cases. Correlation values for RAPD, ISSR and SSR were 0.797, 0.84 and 0.8, respectively, with combined data. ISSR and RAPD exhibited very good correlation value (*r* = 0.7) with each other.

## Discussion

Accurate identification and characterization of different germplasm resources is important for species identification, cultivar development, certification and breeder’s right’s protection (Hale et al. 2006). With the advent of molecular biology techniques, DNA-based markers very efficiently augment morphological, cytological, and biochemical characters in germplasm characterization, varietal identification, clonal fidelity testing, assessment of genetic diversity, validation of genetic relationship, phylogenetic and evolutionary studies, marker-assisted selection, and gene tagging. Owing to plasticity, ubiquity and stability, DNA markers are easier, efficient and less time consuming, especially in perennials where morphological markers are few. The relatively easy to use, low-cost, and highly accurate nature of the polymerase chain reaction (PCR)—based technologies such as RAPD, ISSR, AFLP, and microsatellites are widely appreciated. Although work on morphological characterization of *Zingiberaceous species* has been attempted, its molecular characterization is still in a nascent stage except for some genetic fidelity studies of micropropagated plants and isozyme-based characterization (Sasikumar 2005; Nayak et al. 2005; Mandal et al. 2007).

All the species taken in the present investigation were segregated into two different groups. Interestingly, all species under *Curcuma* and *Zingiber* genus were exclusively grouped under the respective genera. Within the *Zingiber* genus *Z. cassumunar* was found to be isolated from rest of the *Zingiber* species which may be due to its very rare distribution in wild. *Z. officinale* was separated to a distinct clade as it was the only cultivated species. Similarly, *C. longa* was separated from others as it was also a cultivated species.

Chen et al. (1999) used RAPD polymorphism to differentiate within and among *Curcuma wenyujin, C. sichuanensis*, and *C. aromatica*. Kress et al. (2002) studied the phylogeny of the gingers (*Zingiberaceae*) based on DNA sequences of the nuclear internal transcribed spacer (ITS) and plastid *matK* regions. Our result was in agreement with their report that the Zingiber and Curcuma genus were grouped separately. ISSR and SSR markers showed similar grouping with average similarity of 0.311 and 0.296, respectively, whereas RAPD showed less average similarity among species. This could be due to higher polymorphism observed in the above marker. The combined data showed less similarity among species in comparison to SSR and ISSR which might be due to more number of loci observed with RAPD marker. Similar clustering pattern was reflected when correlation was calculated with respect to pooled data versus RAPD, ISSR and SSR markers (0.797, 0.84 and 0.8, respectively).This showed that all the markers were equally effective in characterizing the studied species of Zingiberaceae. A phylogenetic analysis of the tribe Zingibereae (Zingiberaceae) was performed by Ngamriabsakul et al. (2003) using nuclear ribosomal DNA (ITS1, 5.8S, and ITS2) and chloroplast DNA (trnL [UAA] 5′ exon to trnF [GAA]). The study indicated that tribe Zingibereae is monophyletic with two major clades, the *Curcuma* clade, and the *Hedychium* clade. The genera *Boesenbergia* and *Curcuma* are apparently not monophyletic.

Application of PCR-based molecular markers like RAPD, ISSR, SSR and AFLP had been proved by many workers in molecular characterization of different species (Mukerjee et al. 2003; Dikshit et al. 2007; Fu et al. 2008; Lu et al. 2009).

In this report, three PCR-based markers have been used to characterize 10 important species of Zingiberaceae. The uniqueness of the specific bands amplified in different species of Zingiberaceae would help in certification of species as well as in protection of breeder’s right. A correct phylogeny can be established among all constituent taxa of Zingiberaceae through molecular characterization. The potential of the technique will be further realized to fullest extent for identification and tagging of important and novel gene in different taxa, unexplored yet, thus facilitating the conservation and improvement of desired taxa of Zingiberaceae.
